# Barriers and Enablers of Value-Based Procurement in Dutch Healthcare Providers

**DOI:** 10.34172/ijhpm.8514

**Published:** 2025-05-28

**Authors:** Barbara Tip, Niels Uenk, Fredo Schotanus

**Affiliations:** ^1^Coppa Consultancy, Arnhem, The Netherlands.; ^2^Public Procurement Research Centre, Lunteren, The Netherlands.; ^3^Faculty of Law, Economics and Governance, School of Economics, Utrecht University, Utrecht, The Netherlands.

**Keywords:** Value-Based Procurement, Hospitals, Theoretical Domain Framework, Barriers and Enablers, The Netherlands

## Abstract

**Background:**

Value-based procurement (VBP) is gaining traction in healthcare. This approach to procurement prioritizes obtaining the best health outcomes for patients while considering overall healthcare costs. Despite its recognized potential, VBP remains underutilized in hospitals. Little is known about the barriers and enablers of VBP in hospitals. This study aims to identify barriers and enablers specific to VBP in hospital procurement, utilizing the Theoretical Domains Framework (TDF).

**Methods:**

This qualitative study comprises semi-structured interviews with 20 Dutch purchasers working at hospitals. The interviews aim to capture diverse perspectives on VBP, with the data undergoing an extensive coding and analysis process. Using redefined domains of the TDF, themes for barriers and enablers are identified.

**Results:**

We explored the significance of broader barriers and enablers while also pinpointing new and distinctive ones specific to VBP in a hospital context. The newly identified barriers encompass challenges in procurement skills, low strategic priority, environmental context and resources, stakeholder influences, and outcome expectations. Noteworthy barriers include a cost saving focus, resistance to change, influence of the health insurer, and supplier preferences by end-users. Enablers involve stakeholder commitment, positive buyer-supplier relationships, effective storytelling, and demonstrated effectiveness of VBP. Stakeholder influence emerges as an important enabler, emphasizing the importance of the early involvement of medical specialists and other key stakeholders, overcoming resistance and fostering collaboration during VBP adoption in hospitals.

**Conclusion:**

VBP in healthcare prioritizes optimal patient outcomes and value over costs. Although this is a promising concept, we identified several barriers and enablers for implementing VBP. While facing barriers related to procurement skills and environmental context, successful implementation relies on, among other things, training and stakeholder involvement, including early involvement of key stakeholders such as medical specialists and healthcare insurers, ambassadorship, trust-building, and effective storytelling.

## Background

**Key Messages**

**Implications for policy makers**

The study provides valuable insights into the implementation of value-based procurement (VBP) in hospitals:

- Stakeholder involvement, including early involvement of medical specialists and healthcare insurers, ambassadorship, trust-building, and effective storytelling, can enhance the adoption of VBP.
- Healthcare providers could design training programs aimed at enhancing procurement skills specific to VBP.
- Some respondents report hesitation to adopt VBP due to concerns about time consumption. However, those who have implemented VBP actually report time savings. Therefore, allocating additional time and resources for initial VBP implementation is recommended, with the promise of later efficiency gains.
- A revised version of the Theoretical Domains Framework (TDF) specific for healthcare providers can help hospitals identify barriers and enablers for various change and implementation issues.
- VBP strategies need to be adapted to national healthcare systems. In the Netherlands, this could mean shifting insurer-hospital agreements more toward long-term, patient-centered, and outcome-based contracts, better enabling hospitals to procure based on value.

**Implications for the public**

The findings of this study on value-based procurement (VBP) in healthcare have several implications for the public. By emphasizing health outcomes rather than solely focusing on cost, VBP has the potential to improve patient care and make healthcare spending more efficient. However, the study also reveals key barriers that may hinder its adoption, such as limited procurement skills, resistance to change, and a focus on short-term cost savings. Addressing these barriers could lead to better healthcare services and better use of public resources. Furthermore, the involvement of stakeholders, including medical professionals and healthcare insurers, is essential for the successful adoption of VBP. This collaboration can enhance patient care and ensure that the procurement of healthcare goods and services is better aligned with the long-term well-being of patients and the broader public.

Value-based procurement (VBP) has gained significant attention in the healthcare sector, particularly among hospitals.^1^ VBP is an approach to procurement that focuses on maximizing the value delivered to patients by prioritizing health outcomes, while considering overall healthcare costs,^2^ contrasting with traditional procurement, which places greater emphasis on price.^3,4^

 In principle, VBP can be applied to any purchases related to patient health, including buildings or renovations, medical devices, catering services, and more. An example of VBP is an initiative of Guy’s and St Thomas’ NHS Foundation Trust to improve the treatment of lower urinary tract symptoms. The trust piloted a minimally invasive procedure that uses radiofrequency-heated water to reduce prostate size. While the new approach may have involved higher acquisition prices.^5^ The approach drastically reduced operating times and hospital stays.

 In this article, we study the application of VBP by hospitals, which has been shown to bring various benefits. Firstly, it allows hospitals to focus on providing high-quality care to patients, leading to improved outcomes, greater satisfaction among hospitals, and a more effective healthcare system.^6-10^ Secondly, VBP optimizes resource allocation and encourages collaboration among hospitals, suppliers, and stakeholders, facilitating waste elimination and identifying opportunities for innovation and care delivery improvement.^7-9,11,12^

 Extensive research exists on value-based healthcare (VBHC) in general and VBP used by health insurers for contracting healthcare providers. Barriers for health insurers using VBP identified in the literature include relational barriers and resource barriers.^13,14^ Furthermore, in the Netherlands, regulated market mechanisms limit insurers’ ability to engage citizens effectively in procurement processes, undermining their shift towards VBP.^15^ Besides, health insurers face administrative, measurement, and information technology challenges when adopting VBP.^4,16^ Enablers include incentives, measure alignment, provider engagement, performance targets, training, and effective communication.^13^ Besides, open dialogue and collaboration among policy-makers, payers, and providers are important for fostering trust and commitment to VBP initiatives.^17^ Furthermore, the design and a high-intensity of VBP programs also influence their effectiveness.^11^

 However, there is a limited understanding of the application of VBP by hospitals. This gap is unfortunate because there are significant differences between the procurement functions of health insurers and hospitals. Key differences include the stakeholders involved (eg, the role of the medical specialist is significant in hospitals) and the focus on bundled volume purchasing by health insurers compared to individual purchasing processes in purchasing in hospitals.^18^ Furthermore, health insurers typically purchase healthcare as a service from providers, where providers such as hospitals purchase services (also non-medical) as well as tangible products (eg, medical devices, medicine), and real estate. There can also be differences in applicable law and regulations. Moreover, current hospital purchasing decisions often focus on optimizing the individual purchasing process itself, rather than patient outcomes and supplier relationships. For example, currently price or brand preference are often important criteria whereas VBP goes beyond financial aspects to incorporate patient-centered outcomes, satisfaction, and long-term quality.^19^ Due to such differences, it is expected that purchasers working in hospitals face unique barriers and enablers to adopting VBP compared to purchasers working at health insurers. Therefore, the aim of this research is to identify barriers and enablers of VBP in hospitals. Through interviews with purchasers in Dutch hospitals, this study specifically aims to identify barriers and enablers that have not yet been identified in the literature.

###  Conceptual Framework

 To investigate our research question, the Theoretical Domains Framework (TDF) is used as a theoretical framework for understanding the adoption of VBP (See the left hand side of Table 1). The TDF, developed by Michie et al,^20^ consolidates psychological and organizational theories into a structured framework of 15 theoretical domains, each representing key factors influencing behaviour change and implementation. As VBP adoption in hospitals involves a multifaceted process requiring behavioural changes at individual, team, and organizational levels, the TDF’s ability to span these various domains is advantageous.^21^

**Table 1:** The Theoretical Domains Framework Domains and Definitions^21^ and Redefined Domains for Value-Based Procurement

	**Theoretical Domains**	**Definition**	**Redefined Domains for VBP**	**Definition**
1	Knowledge	An awareness of the existence of something	Clinical knowledge	The extent to which healthcare professionals and procurement teams are aware of and understand the clinical evidence, guidelines, and best practices associated with medical products and services.
2	Skills	An ability of or proficiency acquired through practice	Procurement skills	The available skills and expertise by procurement professionals to effectively prepare, select, negotiate, and manage contracts for healthcare products and services.
3	Behavioral regulation	Anything aimed at managing or changing objectively observed or measured actions	Procurement oversight and compliance	The mechanisms for self-regulation and adherence to procurement processes, standards, and regulations to ensure value-based goals are met.
4	Memory, attention, and decision processes	The ability to retain information, focus selectively on aspects of the environment and choose between two or more alternatives	Information processing and decision-making	How information about products and services is collected, analyzed, and used in decision-making processes, with a focus on achieving value-based outcomes.
5	Environmental context and resources	Any circumstance of a person’s situation or environment that discourages or encourages the development of skills and abilities, independence, social competence, and adaptive behavior	Environmental context and resources	The physical and organizational factors in the healthcare environment that impact the ability to procure and deliver VBHC services, including budget constraints and resource availability.
6	Social influences	Those interpersonal processes that can cause individuals to change their thoughts, feeling, or behaviors	Stakeholder influences	The impact of various stakeholders, including clinicians, patients, and suppliers, on healthcare procurement decisions in the context of value-based care.
7	Social/professional role and identity	A coherent set of behaviors and displayed personal qualities of an individual in a social or work setting	Professional roles and stakeholder identities	How healthcare professionals, administrators, and procurement specialists perceive their roles and identities within the context of VBP, and how these perceptions influence decision-making.
8	Beliefs about capabilities	Acceptance of the truth, reality, or validity about an ability, talent, or facility that a person can put to constructive use	Beliefs about procurement abilities	The level of self-assurance and confidence among procurement professionals regarding their ability to secure high-quality and sustainable healthcare products and services while optimizing costs.
9	Optimism	The confidence that things will happen for the best or that desired goals will be attained	Optimism in value-based outcomes	The degree to which healthcare organizations and procurement teams hold a positive outlook on the potential for VBP to improve patient care and cost-efficiency.
10	Intentions	A conscious decision to perform a behavior or a resolve to act in a certain way	Intentions	The organization's and procurement team's plans and motivations for implementing VBP and achieving specific healthcare objectives.
11	Goals	Mental representations of outcomes or end states that an individual wants to achieve	Value-based goals	The specific goals and objectives set by healthcare organizations and procurement teams to promote value, quality, sustainability and cost-effectiveness in healthcare procurement.
12	Beliefs about consequences	Acceptance of the truth, reality, or validity about outcomes of a behavior in a given situation	Beliefs about outcomes	Perceptions of the positive and negative clinical, financial, and patient satisfaction outcomes associated with different procurement decisions.
13	Reinforcement	Increasing the probability of a response by arranging a dependent relationship, or contingency, between the response and a given stimulus	Incentives and disincentives	The use of financial and non-financial incentives or disincentives to influence procurement choices and encourage value-based decisions.
14	Emotion	A complex reaction pattern, involving experiential, behavioral, and physiological elements, by which the individual attempts to deal with a personally significant matter or event	Emotion	The emotional reactions and responses of stakeholders, such as anxiety or enthusiasm, that can influence procurement decisions and relationships.

Abbreviations: VBP, value-based procurement; VBHC, value-based healthcare.

 The TDF facilitates a systematic study of enablers and barriers to VBP adoption by categorizing relevant factors, such as knowledge, skills, beliefs, among others.^20^

 In this article, the TDF domains are redefined to construct a framework more apt for categorizing barriers and enablers for VBP within Dutch hospitals (See the right hand side of Table 1). Customization is needed to ensure that the framework adequately captures the unique factors influencing behaviour change and implementation within the hospital context.

 We proceeded as follows to adjust the domains. First, one researcher redefined the various domains using her extensive knowledge and experience in healthcare procurement. Subsequently, the other two researchers, who also have extensive knowledge and experience in (healthcare) procurement, critiqued the redefined domains and suggested adjustments. Finally, all three researchers engaged in a discussion to reach consensus on the redefined domains. In the end, all the researchers unanimously agreed on the adjustments.

 Within each redefined TDF domain that may influence the adoption of VBP in hospital procurement processes, specific barriers and enablers may be identified.

###  Barriers and Enablers for VBP Plotted in the TDF 

 The literature provides several enablers and barriers for the adoption of VBHC in general and procurement specifically. Adopting VBHC, and also VBP, can be seen as adopting an innovation or something new. Therefore, first the enablers and barriers of innovation adoption will be described. Thereafter, known barriers and enablers will be described for VBP in hospitals.

 Enablers for successful innovation adoption, including VBHC and procurement, encompass policy, oversight, and incentives, planning and change management, resources, environment and culture, execution, and effective communication.^22^ Barriers include a large number of pilot programs without proactive plans for scale and dispersion, additional effort and resources required during implementation, a tendency to revert to old ways when implementation becomes challenging, perception of procurement centred on cost reduction rather than emphasizing long-term value and quality in purchasing decisions, short-term savings targets, lack of knowledge of clinical products, and lack of resources.^22,23^

 In addition to these general barriers and enablers, specific barriers and enablers for VBP in hospitals have been identified in the literature as well. Enablers involve having a clinical leader within hospitals to ensure project success, allocating more decision-making power to the purchasing department, and purchasers with a deep clinical understanding to identify where value can be created.^24^ Barriers include difficulties in translating value into VBP, low organizational or industry maturity with a lack of leadership, and aversion to developing single-source providers.^13^ Furthermore, procurement decisions in hospitals are often influenced by a combination of medical, financial, and strategic considerations. While “on paper” hospital buyers are responsible for procurement, the influence of medical professionals can result in decentralized decision-making by medical staff. Medical professionals purchasing on their own (“maverick buying”) is a common procurement issue.^25^ This can also hinder the implementation of VBP.

 The known barriers and enablers classified within the various domains of the TDF are detailed in Table 2. In the discussion section we explore the differences between the barriers and enablers for health insurers for contracting healthcare providers (identified in the literature) and purchasing in hospitals (mostly identified in our study).

**Table 2:** Barriers and Enablers for Value-Based Procurement in Health Insurers’ Contracting of Healthcare Providers as Identified in the Literature

**Barriers**	**Enablers **
1. Knowledge | Clinical knowledge	
Lack of knowledge of clinical products^23^	Planning and change management^24^
Translation value into VBP^13^	Policy, oversight and incentives^22^
2. Cognitive and interpersonal skills | Procurement skills	
Challenging^22^	Planning and change management^24^
Perception of procurement/myths^13^	
5. Environmental context and resources	
Too many pilot programs^23^	Resources^22,24^
Additional effort and resources^22^	Environment and culture^22,24^
Immaturity of procurement/organization^13^	
Maverick buying^25^	
7. Social/professional role and identity	
Lack of leadership^13^	Execution: Focus, transparency and visibility^24^
8. Beliefs about capabilities | Beliefs about procurement abilities	
Lack of capacity and capability^22^	Effective communication^22,24^
Aversion to developing single-source providers^13^	
11. Goals | Value-based goals	
Narrow price focus^23^	
Short-term saving targets^13^	
14. Emotion	
Lack of trust/mistrust of each other^13^	

Abbreviation: VBP, value-based procurement.

## Methods

 This study utilizes qualitative research to examine VBP barriers and enablers within Dutch hospitals. Interviews serve as the primary method for data collection, as they offer a valuable opportunity to explore and understand the perspectives of hospital purchasers on the subject.^26^ Semi-structured interviews are chosen as the preferred approach due to their flexibility and versatility in gathering in-depth information from participants.^27^ By utilizing this method, we aim to capture nuanced insights into the enablers and barriers associated with VBP in hospitals. We explain the method in more detail below.

###  Dutch Context

 In the Dutch healthcare system, the market operates under a regulated competition model. In this system, health insurers must offer a basic package of healthcare and healthcare insurance is mandatory for all residents. Health insurers contract various healthcare providers, including hospitals. The insurers aim to ensure that all residents have access to necessary care while maintaining healthcare quality and affordability, as well as promoting innovation and preventive measures.

 Healthcare providers, for example hospitals, are paid by the health insurers for the various treatments they perform. How high these payments are depends on the contractual agreement between the healthcare provider and the insurer. These payments are often recorded as a diagnosis treatment combination (DBC). DBC is a healthcare billing system that bundles diagnosis, treatment, and costs into a single payment for a medical service. An example is a patient with a broken leg, where the hospital receives one payment covering the initial consultation, X-rays, surgery, hospital stay, and follow-up care.

###  Semi-structured Interviews

 The participants selected for the interviews were purchasers employed in Dutch hospitals. Informed consent was secured from all participants before their involvement in the study. Participants were provided with detailed information about the study’s purpose, procedures, potential risks, and their right to withdraw at any time.

 A total of 20 purchasers working in four Dutch academic hospitals and sixteen general hospitals were included to provide a diverse range of perspectives and insights. The size of the hospitals varied from approximately 250 to 1350 beds. The working experience of the participants varied from 5 to 30 years. More information about the respondents can be found in Supplementary file 1.

 The interview protocol utilized in this study consisted of a series of open-ended questions. The interview questions were developed by the researchers and some questions where refined based on the first interview. The interview protocol can be found in Supplementary file 2 The duration of the interviews ranged from 45 to 70 minutes.

###  Data Analysis

 All interviews were recorded and subsequently transcribed into text documents. These transcripts then underwent a coding process to identify the barriers and enablers. Initially, the transcripts were read multiple times to establish initial connections and identify relevant codes. Examples of codes used in this study include terms such as “barrier,” “enabler,” “communication problems,” and “policy.” The codes were continuously reviewed by all of the researchers and refined to ensure accuracy and comprehensiveness. After the coding process, the interpretations derived from the interviews were transformed into different themes.

 The primary goal of analysing the transcripts was to explore and identify VBP barriers and enablers. The TDF was used to organize and bring clarity to the findings, rather than to test or validate the TDF itself. The interviews were not conducted with the purpose of confirming or disproving the modified TDF, but rather to generate new insights that are then mapped onto the TDF for better structure and interpretation.

 Relevant data was incorporated into the most appropriate domain code.^21^ After the coding process, the codes and themes were counted and the results underwent multiple rounds of examination and discussion between all of the authors. Subsequently, several barriers and enablers were reclassified, leading to a consensus among the authors. Eventually, unanimous agreement among the authors was reached.

## Results

 In this section, the findings from the interviews are presented. First the barriers are explained followed by the enablers.

###  Barriers

 The analysis of the interviews revealed several barriers that buyers and end-users perceive in relation to the adoption of VBP in hospitals. The overall findings related to the barriers are summarized in Table 3.

**Table 3:** Barriers Mentioned in Interviews

**VBP Domains – Barriers**	**Frequency**
2*. Procurement skills	28
Little knowledge about VBP	8
Limited or lack of ability to persuade stakeholders	8
Buyer lacks sufficient knowledge and experience with VBP	7
VBP assessment matrix is ​​difficult to use	4
Difficult to define adequate project objective in a VBP process	1
5. Environmental context and resources	27
VBP processes take more time	9
The organisation is too immature to adopt VBP	5
Suppliers are too immature	4
Difficult to define value	4
The hospital does not allocate a budget for VBP	3
Insufficient capacity to purchase based on value	2
7. Professional roles and stakeholder identities	15
Traditional view of buyers	6
Unrecognized or poor positioning of the purchasing department within hospital (limited influence on other departments and stakeholders)	2
Difference in knowledge concerning VBP between stakeholders	2
Resistance of stakeholders	2
Buyers must be prepared to relinquish power to other stakeholders	1
Absence of awareness and attention regarding the importance of procurement in realizing savings and adding value	1
Buyer needs to change purchasing behavior	1
6. Stakeholder influences	12
No collaboration between procurement and healthcare sales	4
Supplier preference of end-user	4
Dominant position end-user	3
Lack of intention and motivation from the organization and stakeholders to involve procurement in VBP initiatives	1
12. Beliefs about outcomes	11
Procurement should focus on savings	7
Too many objectives in an VBP project which makes it too complex	3
Difficult to define the benefits for patients	1
13. Incentives and disincentives	10
Financial structure of the hospitals and health insurers; little to no budget for VBP	8
No incentive to involve purchasing	2
10. Intentions	9
VBP is not a priority for hospitals	9
4. Information processing and decision-making	5
Lack of best practices of VBP	2
Too many stakeholders	1
Traditional view from healthcare	1
Too little knowledge about VBP	1
9. Optimism in VBP	4
Labelling the project as VBP is a hindrance to the process and counteracts its effectiveness	2
Misunderstanding within the organization	2
14. Emotion	4
Lack of trust in procurement among stakeholders	2
Fear for the unknown	2
1. Clinical knowledge	1
No knowledge about DBC making it challenging to persuade the health insurers	1
11. Value-based goals	1
Buyer lacks ownership	1
3. Procurement oversight and compliance	1
Procurement rules make it difficult to apply VBP	1
8. Beliefs about procurement abilities	1
Afraid of making mistakes; risk aversion	1
**Total**	**129**

Abbreviations: VBP, value-based procurement; DBC, diagnosis treatment combination. * The numbers refer to the original domains listed in Table 1.

 The most frequently mentioned barriers fall into the categories procurement skills and environmental context and resources. We discuss these domains and a few other notable barriers below.

###  Procurement Skills Barriers

 The majority of respondents perceive and experience procurement skills often as a barrier to adopting VBP in hospitals. This can be explained by the fact that VBP involves shifting from traditional procurement practices to prioritizing quality, outcomes, and patient-centric approaches, which demands a different skill set. Interestingly, procurement skills are not mentioned as an equally significant barrier by all respondents. This issue is more frequently noted in smaller hospitals compared to larger ones, and it is also more commonly observed in general hospitals than in academic hospitals.

 Furthermore, several respondents expressed a lack of in-depth knowledge about the specifics of VBP and how to adopt it. Without a clear understanding of what VBP entails, it is challenging for buyers to transition to VBP.

 Convincing stakeholders about the value of VBP requires purchasers to communicate effectively across different domains and cater to the interests and concerns of each group. Besides that, hospitals often have established practices and hierarchies that resist change and stakeholders have diverse priorities. Convincing stakeholders to embrace VBP might face resistance due to ingrained systems, historical procurement approaches, or a reluctance to deviate from familiar practices.

 It is noteworthy that respondents with most procurement experience identify a greater number of barriers related to procurement skills. This may reflect their awareness of the challenges inherent in engaging with diverse stakeholders. Experienced respondents emphasize that their limited ability to persuade stakeholders effectively is a significant barrier to VBP. One respondent, with more than 15 years’ experience, mentioned: “*You have so many stakeholders involved. […]—everyone has an opinion. Then there are various supporting departments, such as clinical physics. It becomes very challenging when they are involved early on, having a say in deciding which method to use.”* In contrast, respondents with less than 15 years of procurement experience did not express similar concerns. This underscores the importance of developing both procurement skills and stakeholder engagement strategies to facilitate VBP.

 In addition to the barriers of convincing stakeholders and limited knowledge about VBP, the complexity of the VBP assessment matrix is mentioned exclusively by respondents with over 15 years of purchasing experience, most of whom have not implemented a VBP project. *“It seems challenging to determine which provider is the best based on the assessment matrix.” *Their experience might have made them more aware of the complexities and challenges associated with integrating new methodologies.

###  Environmental Context and Resources Barriers

 In addition to barriers associated with procurement skills, many respondents identify barriers related to resource and environmental considerations. These factors are mentioned more frequently by larger hospitals compared to smaller ones. Furthermore, respondents from academic hospitals report them most often. Larger and academic hospitals may encounter more complex operational demands and greater regulatory oversight, which may heighten their awareness of resource constraints and environmental factors. In contrast, general and smaller hospitals might face more straightforward challenges related to resource allocation.

 More broadly, the frequent mention of resource and environmental considerations can be attributed to the specific nature of hospitals. Hospitals often operate under budget pressure, which can limit investments in VBP. Additionally, the hierarchical and multi-layered structures in hospitals can sometimes hinder fast decision-making or flexible procurement. Several respondents also express concerns that VBP demands too much time, which they lack. Remarkably, respondents who have not yet implemented expect it to be very time-consuming.

###  Other Notable Barriers 

 We also identified several specific but still noteworthy barriers. One of these relates to strategic intentions: namely, that VBP is not a priority. This barrier is frequently cited by respondents who have not yet implemented VBP. This lack of prioritization might stem from resource allocation, competing projects seen as more urgent or impactful, resistance to change, social factors, or an organizational culture that does not fully embrace VBHC.

 Another frequently mentioned barrier is a focus on cost savings. When a procurement department focuses on savings, adopting VBP presents challenges due to a shift in procurement philosophy. A respondent mentioned: *“Rigorous savings targets and price-centric focus shape the procurement department’s behaviour. This leads the buyers to focus more on achieving the lowest price.”* Cost-saving strategies predominantly aim at obtaining lower prices. A shift to VBP would necessitate a broader evaluation of suppliers based on their capacity to add value. Finally, it is noteworthy that achieving savings is primarily cited as a barrier by respondents who have not yet adopted VBP.

 Brand preference is also a specific barrier to adopting VBP. As one respondent explained: *“Resistance and preferences from departments and end users for certain brands and suppliers complicate the process of aligning all stakeholders and initiating VBP.”* This resistance is influenced by factors such as medical training, personal inclinations, interactions during supplier visits, or even sponsorships. Brand loyalties or personal inclinations pose a challenge for buyers aiming to implement VBP in hospitals. It requires strategies that not only emphasize the value of alternative products but also address the underlying reasons behind stakeholders’ preferences.

 Another specific and notable barrier is the financial structure of the hospitals and healthcare insurers. While insurers could support VBP in hospitals by sharing detailed performance data benchmarked against similar institutions, enabling the identification of areas for improvement, respondents mentioned that insurers had a negative impact on VBP in hospitals. Insurers often prioritize cost reduction when negotiating contracts, which may conflict with the emphasis on value in VBP. Additionally, the perceived inflexibility in contract negotiations imposed by insurers can hinder hospitals’ ability to adopt VBP. Furthermore, limited or insufficient reimbursement for services related to VBP initiatives creates disincentives for hospitals to invest resources in these value-centric methodologies. One interviewee observed: *“I’ve noticed that healthcare insurers reimburse for certain products and services, but they are less likely to cover the more expensive ones that offer greater value.” *Interestingly, the financial structure within Dutch healthcare is primarily mentioned as a barrier by respondents who had already implemented VBP. This highlights a tension between the incentives created by the Dutch financial structure and the principles of VBP. One respondent, who initiated a VBP project but had to discontinue it, remarked: *“If patients return less frequently, we receive less reimbursement. It was not a positive business case for the hospital.” *Another respondent had a similar experience with their VBP project: *“It didn’t get off the ground due to the hospital reimbursement system. The health insurer indirectly incentivizes hospitals to encourage patients to return more frequently to the hospital […].” *

 Finally, it is noteworthy that outcome expectations are primarily cited as a barrier by respondents who have not adopted VBP. They express difficulty in defining the benefits for patients, as well as concerns about having “too many objectives” within their VBP projects.

###  Enablers

 The analysis of the interview data also highlighted several enablers for VBP. Respondents who have already adopted VBP mentioned more enablers, as we will describe in more detail later in this section. The overall findings related to the enablers are summarized in Table 4.

**Table 4:** Enablers Mentioned in Interviews

**VBP Domains – Enablers**	**Frequency**
6*. Stakeholder influences	**19**
Good buyer-supplier relationship	5
Strong willingness of the project owner facilitates implementation of VBP	4
Ensure good communication with the stakeholders	3
The ability to gain trust	2
Good collaboration between procurement and healthcare sales	2
Stakeholders commitment to apply VBP	1
Supplier preference of end-user	1
Let the doctors think they came up with it themselves	1
7. Professional roles and stakeholder identities	**19**
Get stakeholders onboard	8
Identify your stakeholders	4
You need an ambassador who understands the concept and discusses it with other departments	3
Presence of an integral project leader	1
Project team with all the relevant stakeholders	1
Involve a limited number of stakeholders	1
Position of the purchasing department within the hospital	1
2. Procurement skills	**12**
The ability to convince stakeholders	3
Be open to VBP	2
Have experience with VBP	2
Don't put too much emphasis on risks	1
Show the possibilities of VBP	1
Define good assessment criteria	1
Use a limited number of requirements	1
Make sure you are well prepared	1
1. Clinical knowledge	**11**
Understand that you are purchasing an outcome	3
Ensure physical proximity among all stakeholders	3
Understand VBP well	2
Knowledge of the market	1
Be able to define the need	1
It must be proven effective	1
9. Optimism in VBP outcomes	**7**
Sharing best practices	3
Show that VBP is an innovation	1
Show value for the patient	1
Involve patients	1
Promote VBP often	1
5. Environmental context and resources	**9**
Make time for VBP	3
Use the framework of VBP	2
Take small steps	2
You need an ambassador who understands VBP and discusses it with other departments	1
Be close to the carepath	1
11. Beliefs about outcomes	**5**
VBP requires less time compared to drafting more traditional technical requirements	2
As a buyer, you must be willing to invest a lot upfront before seeing actual results	1
Alignment with organizational goals	1
Link QALYs to VBP	1
8. Beliefs about procurement abilities	**2**
As a buyer, take on the role of a process supervisor rather than a project leader	2
4. Information processing and decision-making	**2**
You really need to come up with a compelling story	1
Using patient data instead of involving actual patients sometimes works better in a VBP project	1
13. Incentives and disincentives	**2**
Competition stimulates suppliers	1
Alignment with organizational goals	1
3. Procurement oversight and compliance	**1**
No strict procurement rules allow for more creativity and space to implement VBP	1
11. Value-based goals	**1**
Use the ideas from the hospital	1
10. Intentions	**0**
14. Emotion	**0**
Total	**91**

Abbreviations: VBP, value-based procurement; QALYs, quality-adjusted life years. * The numbers refer to the original domains listed in Table 1.

 The most frequently mentioned enablers are in the category stakeholder influences and professional roles and stakeholder identities. We discuss these domains and a few other notable enablers in more detail below.

###  Stakeholder Influence Enablers

 Most respondents perceive stakeholder influences as an enabler to adopting VBP in hospitals. However, it is noteworthy that resistance from various stakeholders is also commonly cited as a significant barrier. The prominence of stakeholder influences being perceived as an enabler aligns with the understanding that the active involvement of stakeholders is imperative for VBP adoption. This interplay between stakeholders’ influence as both an enabler and a barrier signifies the importance of stakeholder engagement and collaboration for VBP adoption. During the interviews, respondents frequently mentioned that the supplier is an important stakeholder as well and a good relationship with suppliers is significant. One interviewee explained,* “Building a strong partnership between buyers and suppliers is important for VBP. It’s not just about transactions, but about collaboration that drives mutual value creation and long-term success.” *A good relationship also promotes shared responsibility for outcomes, emphasizing accountability and commitment toward achieving desired results within a VBP framework. Additionally, a strong buyer-supplier relationship encourages long-term partnerships built on trust and reliability, supporting continuous improvement and growth over time.

 Small and medium-sized hospitals more often emphasized stakeholder influences. This variation can be attributed to the differing operational scales and complexities of larger and smaller hospitals. Larger hospitals typically have more resources and specialized staff, allowing them to engage with a diverse array of stakeholders.

###  Professional Roles and Stakeholder Identities Enablers

 In the category professional roles and stakeholder identities, getting stakeholders onboard is the enabler which is most frequently mentioned by the respondents. Involving stakeholders serves several purposes. Firstly, their active participation ensures collective support and commitment to the transition towards VBP. Moreover, engaging stakeholders aligns diverse perspectives and objectives towards a unified goal of prioritizing value in procurement decisions. Furthermore, proactive stakeholder engagement helps in addressing potential resistance or challenges that might arise while adopting VBP. By involving stakeholders early on and addressing their concerns, buyers can navigate potential hurdles more effectively. During the interviews different ways of getting the stakeholders onboard were mentioned. They frequently mentioned enthusiasm to engage stakeholders and the use of storytelling to illustrate the benefits and impact of VBP. As one interviewee stated, *“Make sure you have a clear understanding of your stakeholders and get them excited about your project.” *By sharing stories of successful purchases, professionals can inspire and motivate others to embrace this approach. Storytelling also helps create a sense of connection and empathy among stakeholders. When individuals within the hospital setting hear stories of how VBP has improved care, they better understand the importance of VBP.

 Another important enabler that has influence on getting stakeholders onboard is the demonstration of the effectiveness of VBP. Participants emphasized that stakeholders are more likely to be on board when the cost-effectiveness of VBP has been proven: *“You have to conduct a cost-benefit analysis and compare the cost-effectiveness of VBP with traditional procurement methods.”*

 Taking small steps and building trust through the initiation of small initiatives were also identified as enablers. Participants highlighted the significance of gradually adopting VBP and creating trust among stakeholders. One respondent mentioned: *“It’s important to take small steps and create trust and start with little initiatives.” “When there is no trust between the stakeholders, the process is guaranteed to go wrong.” *

 When comparing larger and smaller hospitals, small and medium-sized hospitals more often emphasized professional roles and stakeholder identities as an enabler. Similar to stakeholder influences, this variation can be attributed to the differing operational scales and complexities of larger and smaller hospitals.

###  Other Notable Enablers 

 It is noteworthy that respondents highlight how VBP saves time, while others indicate it consumes a significant amount of time, as previously mentioned in the barriers. The time factor seems to depend on the buyer’s skills and experience—whether it incurs or saves time. The implementation of VBP might demand more time due to the complexities of evaluating suppliers based on value criteria. However, when procurement professionals become more familiar with VBP, efficiency gains can emerge over time. Improved skills and familiarity with VBP can ultimately lead to quicker decision-making and more efficient supplier partnerships. Experienced buyers tend to navigate VBP processes more efficiently. However, those lacking expertise in VBP might initially invest more time in learning to apply VBP.

 During the interviews it also became clear that ambassadorship can serve as an enabler for VBP within hospitals in several ways. A respondent mentioned: *“An ambassador who understands the concept and can engage in discussions with other departments is essential.”* Firstly, when healthcare professionals and staff members become ambassadors for VBP, they actively promote and advocate for its implementation. They can engage in educational initiatives to raise awareness about the benefits of VBP, encouraging others to adopt this approach. It can also create a culture of collaboration and teamwork. When individuals within the organization take on the role of ambassadors, they foster a sense of shared responsibility and commitment towards VBP goals. This can lead to increased cooperation among different departments and stakeholders, resulting in more effective and efficient procurement.

 Clinical knowledge and procurement skills are more frequently mentioned as enablers by respondents who have implemented VBP. *“It is important to have a deep understanding of the content and to be well-acquainted with the process. When you enter the organization with substantial knowledge, you will find that people are more willing to cooperate.”*

 Optimism regarding VBP outcomes is predominantly expressed by respondents who have not yet engaged in the process. They also have practical suggestions. A respondent mentioned: *“I think it is beneficial to have a kind of benchmark and best practices from other hospitals. There is much to learn from this, and it may help in convincing the organization.”*

## Discussion

 The main research question of this article is to identify barriers and enablers to VBP in a hospital context. We used the TDF framework to categorize the barriers and enablers. We had to make adjustments to the TDF domains to have a better fit with the hospital and procurement context. The modified framework turns out to be effective in subdividing and better understanding the barriers and enablers in this context.

 In the remainder of this section, we discuss our contributions to the understanding of barriers and enablers. Next, we address the practical and policy implications, the limitations of our research, as well as suggestions for further research.

###  Barriers 

 Our findings highlight that hospitals face some common, but also several distinct challenges in adopting VBP, expanding the literature beyond insurer-led approaches to focus on hospital-based procurement. In this research, respondents confirmed some barriers to VBP in the hospital context that are also noted in the literature, such as lack of knowledge, limited resources, difficulty in translating value into VBP, short-term savings targets, and low organizational maturity.^22,23^ Additionally, we identified several new findings. One key contribution is that respondents who had not yet completed a VBP project reported a wider range of barriers compared to those who had successfully implemented VBP. This discrepancy may be attributed to their lack of firsthand experience, which can foster uncertainty. Addressing these concerns through educational initiatives, open communication, and the sharing of success stories may help mitigate uncertainty and enhance confidence in the VBP process.

 This research also provided new insights about barriers to VBP in different hospital contexts. Notably, large hospitals are less likely to view procurement skills as a barrier compared to small and medium-sized hospitals. Larger hospitals typically face more complex procurement needs and usually have more specialized staff and resources, which can lead to less challenges in developing procurement skills. They may also have more experience with alternative procurement procedures required for complex purchases, which are less common in smaller hospitals, where specifications may be less clearly defined by the hospital itself. The procurement skills needed for these flexible, more goal oriented purchases are likely also valuable for adopting VBP. In contrast, resource and environmental considerations, an often mentioned barrier, is more frequently noted by larger hospitals compared to smaller hospitals. Furthermore, respondents from academic hospitals reported resource and environmental-related barriers most often, likely due to the complexity of their operations and heightened regulatory oversight they experience, which increases their awareness of these challenges. The barriers regarding resource considerations within VBP in hospitals cover a broad spectrum. Hospitals not only face budget constraints but also operational complexities of hierarchical structures and patient-centric decision-making. These complexities distinguish VBP in hospitals from the more mainstream VBP practices in healthcare insurers.

 Furthermore, it is noteworthy that experienced respondents identified the complexity of the VBP assessment matrix as a barrier, a concern not raised by respondents with less purchasing experience. More experienced purchasers prolonged exposure to traditional procurement frameworks may also have heightened their scepticism and concern regarding the practical application of VBP principles, leading them to articulate barriers more readily than less experienced respondents who may not fully grasp the intricacies involved.

 In addition, respondents lacking experience with VBP often perceive outcome expectations as a barrier. This perception may arise from their uncertainty regarding the anticipated benefits of VBP, leading to concerns about potential outcomes. In contrast, those who have successfully implemented VBP tend to have firsthand knowledge of its advantages, thereby reducing the perceived significance of these expectations as barriers.

 Another finding is related to the role of health insurers as the financer of hospitals. Health insurers apply VBP within their own domain. Therefore, one might expect them to encourage its adoption within hospitals, yet this is not perceived by the respondents. The health insurers often still focus on the lowest price. What we observe here is not unique. Issues often arise in situations of managed competition where the interests of insurers, hospitals, and patients are not optimally aligned. This is evident, for example, in the lack of reimbursements for new treatments that are deemed valuable by both patients and healthcare professionals, yet are not covered by insurers, often due to the need for further evidence.^28^

 During the interviews, all respondents identified more barriers than enablers to VBP. Notably, respondents who had engaged in a VBP process reported an almost equal number of barriers as enablers. This suggests that those who have not undertaken a VBP initiative perceive a greater number of barriers than their counterparts. It raises the question of whether the perceived barriers may be less significant than initially thought.

 When comparing responses between academic hospitals and general hospitals, there were almost no differences in the frequency with which barriers and enablers were mentioned. This suggests that the type of hospital—whether academic or general—does not influence the perceived balance of barriers and enablers within these environments. It may be inferred that both types of institutions face mostly similar structural or systemic challenges, or that most differences in operational complexity between academic and general hospitals are not sufficient to alter these perceptions. This finding aligns with previous studies on VBHC.^29^

 However, when hospital size is considered, a difference emerges. Respondents from smaller hospitals reported experiencing more barriers compared to those working in medium-sized and larger hospitals. This suggests that hospital size may play a role in shaping the organizational capacity to manage VBP barriers. Smaller hospitals may face resource constraints, including limited staffing, restricted financial flexibility, and reduced access to advanced technologies or specialized departments, which could increase the perception of VBP barriers. Moreover, the smaller scale of operations might increase challenges related to workflow, adaptability, and the implementation of best practices, as smaller hospitals may lack the economies of scale.

 The observed differences in perceived barriers across hospital sizes could also reflect differences in culture, leadership, and support systems. Larger hospitals might have more robust administrative structures, dedicated quality improvement teams, and access to external partnerships or networks, which could facilitate problem-solving and mitigate perceived barriers. In contrast, smaller hospitals might operate with leaner management structures, resulting in more operational burdens on individual staff members, thereby intensifying the perception of barriers.

 These findings underscore the importance of considering hospital size as a factor in both the identification of barriers and the development of tailored interventions. Our data suggests that while hospital type (academic versus general) does not appear to substantially influence the perception of barriers, the scale of operations does, and this could be taken into account in future research and policy development. Addressing the unique challenges faced by smaller hospitals may require targeted support and resources, such as strengthening managerial capacity, providing access to external expertise, and offering financial or technical assistance programs.

 Finally, the barriers can also enable the adoption of VBP when they are addressed strategically. For example, challenges such as limited procurement skills might become enablers if organizations invest in training and capacity building to strengthen procurement expertise. By reframing barriers as opportunities for improvement, they can help facilitate rather than hinder the adoption of VBP.

###  Enablers

 The more specific enablers for VBP which are mentioned in the literature are having a clinical leader within hospitals to ensure project success, allocating more decision-making power to the purchasing department, and (procurement) managers with a deep understanding of clinical processes and technology throughout the care cycle to identify areas where value can be created.^23^ All the above mentioned enablers are mentioned by the respondents with the exception of technology understanding throughout the care cycle. In the current context studied in the Netherlands, it seems that technology understanding is no longer seen as a distinguishing barrier or enabler for VBP. Nevertheless, clinical knowledge and procurement skills are more frequently mentioned as enablers by respondents who have implemented VBP. This suggests that those with practical experience recognize the importance of understanding clinical evidence and possessing the necessary skills to effectively navigate the procurement process.

 The new enablers identified in this research focus more on engaging stakeholders and professional roles alongside stakeholder identities. When VBP is implemented, stakeholders, such as medical specialists, wield considerable influence over the process. The resistance among medical professionals and the intricate web of relationships among stakeholders, for example the medical specialist and the supplier, significantly impacts procurement decisions within hospitals. Hence early involvement of medical specialists is important. Moreover, it is notable that several respondents refrain from adopting VBP because they fear it might consume significant time. Conversely, respondents who have implemented VBP indicate that it actually saves them time, for instance, because they are not bound by stringent sets of requirements.

 Small and medium-sized hospitals more often emphasized stakeholder influences as an enabler. Small and medium-sized hospitals may have less access to specialized procurement knowledge, making stakeholder influences a more prominent enabler. These hospitals may rely more on existing relationships with stakeholders to drive VBP initiatives due to their limited structural support. Interestingly, we did not observe any differences between academic and general hospitals in this regard. The absence of significant differences between academic and general hospitals suggests that both operate under similar pressures regarding VBP adoption.

 When comparing larger and smaller hospitals, small and medium-sized hospitals more often emphasized professional roles and stakeholder identities as an enabler. As discussed before, this variation can be attributed to the differing operational scales and complexities of larger and smaller hospitals.

 Optimism regarding VBP outcomes is predominantly expressed by respondents who have not yet engaged in the process. For the respondents who have not yet engaged in VBP, it is important to further convince the organization of the significance of VBP by performing benchmarks and learn from other organisations.

###  Practical and Policy Implications

 Addressing barriers to VBP implementation requires a multifaceted approach. Training programs could help, as they address barriers like limited procurement skills related to VBP and low strategic prioritization. By enhancing procurement expertise, hospitals can navigate the complexities of VBP more effectively. Sharing best practices for evaluation mechanisms may also help to improve the effectiveness of VBP.

 The early involvement of medical professionals and other key stakeholders is also important. Given their influence on procurement decisions, engaging them early in the VBP process helps mitigate resistance and ensures alignment with clinical needs.

 Smaller hospitals may benefit from additional support in resource allocation to counter the perception of resource constraints. They could also consider joint procurement to increase the pool of available resources.

 To counteract perceptions that VBP requires excessive time investments, it is important to communicate the long-term efficiencies VBP can offer, thereby showing its practical benefits.

 Furthermore, practitioners can use the modified TDF to identify the specific barriers and enablers relevant to a particular hospital’s context. Based on the enablers and barriers identified in our study, they can tailor VBP strategies to their procurement structure.

 Finally, we examine in more detail the policy implications related to the role of health insurers, identified as a barrier in our study, and explore the differences between VBP in the US and the Dutch healthcare system. The concept of VBP was developed in the US with a specific focus on insurers contracting healthcare providers. As noted in our literature review, most research has focused on this aspect. Our study, however, explores VBP in a different context—a Dutch one—where it applies not to the relationship between healthcare providers and insurers but to the relationship between healthcare providers and their suppliers.

 The US healthcare system differs in several aspects from the Dutch system. In the US, cost-effectiveness and cost-utility analyses are not permitted in Medicare and Medicaid policy decisions due to ethical and legal concerns over cost-based rationing.^28^ Instead, the Centers for Medicare & Medicaid Services have introduced VBP initiatives, such as the Hospital VBP Program, which financially rewards hospitals based on quality metrics rather than volume.^16^ Private insurers and research institutions in the US do conduct cost-effectiveness analyses, though these do not directly influence federal reimbursement policies.

 By contrast, in the Netherlands, cost-effectiveness and utility analyses, combined with willingness-to-pay thresholds, are primarily used at the national level to determine which treatments receive public funding.^15^ This approach allows policy-makers to make reimbursement decisions across healthcare, long-term care, and preventive care. As a result, at the hospital level, procurement decisions made by health insurers often focus on price negotiations within predefined DBCs.^18^ While the Dutch system has several benefits, our study finds that it also creates barriers for hospitals’ procurement strategies. The emphasis on price negotiations by insurers in many instances reinforces a similar price-driven approach within hospitals, limiting the extent to which VBP is adopted by hospitals.

 In the US, VBP helps compensate for the absence of centralized cost-effectiveness evaluations, whereas in the Netherlands, its role remains less clearly defined. Our research highlights the importance of reducing the emphasis on price in negotiations between insurers and hospitals, enabling hospitals to make more value-driven procurement decisions.

 To ensure that VBP better aligns with Dutch healthcare policies, we propose the following policy changes. Unlike in the US, where VBP improves efficiency in a fragmented system and operates in the absence of centralized cost-effectiveness evaluations, in the Netherlands, it could complement rather than replace cost-effectiveness and utility analyses. This could be achieved by making agreements between hospitals and insurers more long-term, patient-centered, and outcome-oriented. Some steps in this direction have already been taken, such as bundled payments and some shared savings models. However, further development is needed to scale these initiatives. For example, hospitals could be financially rewarded if they can demonstrate that they procure and deliver care based on value rather than price alone. Strengthening such mechanisms could help remove one of the barriers to the adoption of VBP by hospitals.

###  Limitations and Future Research 

 This study has several limitations, which also provide a basis for future research. First, while the sample selection and recruitment process contribute to the overall trustworthiness of the findings, the characteristics of the sample may limit the breadth of perspectives captured. The research employed an exploratory and qualitative approach, focusing specifically on Dutch procurement professionals. Consequently, the sample size and composition may not fully represent the diverse experiences and viewpoints related to VBP. Additionally, the exclusion of other stakeholders, such as end-users involved in the procurement process, means that the identified barriers and enablers primarily reflect the procurement perspective. Future research could address other perspectives as well.

 Future research employing a quantitative approach that targets a larger and more diverse group of respondents to survey views and experience of VBP, could complement this qualitative research. It is also relevant to note that this research was conducted in the Netherlands. Therefore, while the results provide valuable insights, they may not be directly applicable to other countries without further validation. However, many identified barriers and enablers are likely to resonate with hospitals in other developed nations, particularly those with similar healthcare systems and economic conditions. Furthermore, research into which outcome measurement criteria best align with both clinical and procurement objectives could help address the need for universally accepted metrics for evaluating value. Finally, future research could also examine how hospitals in other (European) healthcare systems and the US implement VBP and to what extent, for example, regulated reimbursement models influence adoption.

## Conclusion

 VBP is a concept that has gained increasing attention in the healthcare sector in recent years. VBP emphasizes the importance of obtaining the best possible health outcomes for patients while considering the overall costs of healthcare provision. However, the implementation of VBP in healthcare faces several barriers and enablers.^3^

 In our study, we investigated barriers and enablers specific to VBP within the hospital context. We explored the significance of broader barriers and enablers while also pinpointing new and distinctive ones specific to VBP in hospitals. The newly identified barriers primarily revolve around procurement skills, low strategic priority, and resource and environmental context. The barriers regarding the resource considerations within VBP in hospitals cover a broader spectrum than procurement, such as budget constraints and hierarchical structures.

 The new enablers are more related to stakeholder involvement. When VBP is implemented, stakeholders, such as medical specialists, wield considerable influence over the process. Resistance among them could significantly impact procurement decisions within hospitals. Hence, it is important to ensure early involvement of medical specialists and to invest in trust-building, ambassadorship, storytelling and support creation.

 Moreover, it is notable that several respondents refrain from adopting VBP because they fear it might consume significant time. Conversely, respondents who have implemented VBP indicate that it actually saves them time. Another remarkable finding is related to the role of health insurers as the financer of hospitals. They do not encourage its adoption within hospitals, according to the respondents. Finally, it is interesting that in the current context studied in the Netherlands, it seems that technology is no longer seen as a distinguishing barrier or enabler for VBP.

 Another finding in our study was the variation in barriers and enablers to VBP adoption based on hospital size. Smaller hospitals tended to report more barriers related to limited resources and procurement expertise, potentially due to, among other things, constraints in staffing and access to specialized knowledge. In contrast, larger hospitals more frequently cited challenges related to hierarchical structures and resource management, which may arise from more complex organizational dynamics.

 Respondent experience with VBP also influenced perceptions of barriers and enablers. Respondents with limited VBP experience expressed more concerns regarding resource demands, required time, and perceived complexity, which could reflect uncertainty about VBP. Conversely, experienced respondents were more likely to identify specific operational barriers, such as the complexities of the VBP assessment matrix.

 While overcoming the barriers and using the enablers identified, hospitals could more often apply VBP. The goal of a purchasing department within a hospital could change then more often from realizing savings to increasing value. By prioritizing value over cost alone (essential to VBP), hospitals can make more informed purchasing decisions that result in better patient outcomes and more efficient use of resources.^7-10^

## Ethical issues

 This study complies with the Research Data Management Policy of the Faculty of Law, Economics, and Governance (LEG) at Utrecht University, as established by the BOS meeting on September 11, 2019. The interviews were conducted by a consultant from the advisory firm Coppa. The study received ethical approval from the company, although the company does not issue reference numbers. It is a minimal-risk research study. No patient-sensitive or personal data were collected. Researchers contacted interview participants in writing, outlining the purpose of the study and the interview process. Informed consent was obtained from each participant prior to the interview. Participants were assured of confidentiality and anonymity, that participation was voluntary, and that they were free to withdraw from the study at any time. No participants withdrew their consent.

## Conflicts of interest

 Authors declare that they have no conflicts of interest.

## Supplementary files


Supplementary file 1. Respondents.

Supplementary file 2. Interview Protocol.
