# Supplementary material for: Barriers and Enablers of Value-Based Procurement in Dutch Healthcare Providers
Source: Int J Health Policy Manag. 2025 May 28;14:8514. doi: 10.34172/ijhpm.8514 (PMC12337166; doi:10.34172/ijhpm.8514)
Supplement: Supplementary file 1 — Respondents. [file ijhpm-14-8514-s001.pdf]

Article title: Barriers and Enablers of Value-Based Procurement in Dutch Healthcare Providers
Journal name: International Journal of Health Policy and Management (IJHPM)
Authors' information: Barbara Tip[1]*, Niels Uenk[2], Fredo Schotanus[3]
[1]Coppa Consultancy, Arnhem, The Netherlands.
[2]Public Procurement Research Centre, Lunteren, The Netherlands.
[3]Faculty of Law, Economics and Governance, School of Economics, Utrecht University, Utrecht, The Netherlands.
***Correspondence to:** Barbara Tip; Email: Barbara.tip@coppa.nl
Citation: Tip B, Uenk N, Schotanus F. Barriers and enablers of value-based procurement in Dutch healthcare providers. Int J Health Policy Manag. 2025;14:8514. doi:10.34172/ijhpm.8514

Supplementary file 1. Respondents

Respondent	Job title	Hospital	Size based on the number of beds	Working experience	Experience with VBP
1	Purchasing manager	General	Medium	15 – 30 years	Yes
2	Purchasing Manager	Academic	Small	15 – 30 years	Yes
3	Purchasing Manager	Academic	Large	15 – 30 years	No

4	Purchasing Manager	General	Medium	15 – 30 years	Yes
5	Head of purchasing	General	Small	6 – 14 years	No
6	Purchasing Manager	General	Medium	6 – 14 years	No
7	Purchasing Manager	General	Medium	15 – 30 years	No
8	Purchasing Manager	General	Medium	6 – 14 years	No
9	Purchasing Manager	Academic	Large	6 – 14 years	No
10	Purchasing Manager	General	Large	6 – 14 years	Yes
11	Strategic buyer	General	Medium	6 – 14 years	No

12	Strategic buyer	General	Large	6 – 14 years	Yes
13	Head of purchasing	General	Small	0 – 5 years	No
14	Purchasing Manager	General	Small	15 – 30 years	Yes
15	Purchasing Manager	General	Small	15 – 30 years	Yes
16	Purchasing Manager	General	Large	15 – 30 years	Yes
17	Purchasing Manager	General	Small	15 – 30 years	No
18	Head of purchasing	Academic	Medium	6 – 14 years	No
19	Purchasing Manager	General	Large	15 – 30 years	Yes
20	Strategic buyer	General	Small	0 – 5 years	No
