# Supplementary material for: Barriers and Enablers of Value-Based Procurement in Dutch Healthcare Providers
Source: Int J Health Policy Manag. 2025 May 28;14:8514. doi: 10.34172/ijhpm.8514 (PMC12337166; doi:10.34172/ijhpm.8514)
Supplement: Supplementary file 2 — Interview Protocol. [file ijhpm-14-8514-s002.pdf]

Article title: Barriers and Enablers of Value-Based Procurement in Dutch Healthcare Providers
Journal name: International Journal of Health Policy and Management (IJHPM)
Authors' information: Barbara Tip[1]*, Niels Uenk[2], Fredo Schotanus[3]
[1]Coppa Consultancy, Arnhem, The Netherlands.
[2]Public Procurement Research Centre, Lunteren, The Netherlands.
[3]Faculty of Law, Economics and Governance, School of Economics, Utrecht University, Utrecht, The Netherlands.
***Correspondence to:** Barbara Tip; Email: Barbara.tip@coppa.nl
Citation: Tip B, Uenk N, Schotanus F. Barriers and enablers of value-based procurement in Dutch healthcare providers. Int J Health Policy Manag. 2025;14:8514. doi:10.34172/ijhpm.8514
Supplementary file 2. Interview Protocol

General questions

1. Which organization do you work for?
2. What is your position within the organization?
3. How long have you been employed in this role?

Procurement-related questions

4. How many procurement professionals are in your department?
5. What types of products or services are typically procured?
6. What is the annual procurement volume for your organization?
7. What specific categories of products or services do you primarily purchase?

Value-Based Procurement (VBP)

8. Are you familiar with VBP?
9. How did you become acquainted with the concept of VBP?

10. Is VBP implemented within your organization?

11. What objectives do you aim to achieve through VBP?

12. How would you define VBP?

13. What challenges do you encounter regarding the implementation of VBP?

14. How many VBP projects have you completed?

15. Which specific projects have you undertaken?

16. Who were the key stakeholders involved in these projects?

17. What outcomes were achieved from these projects?

18. Which factors within the healthcare system influence your efforts related to VBP?

19. What obstacles make it difficult to implement VBP?

20. What barriers do you experience in relation to VBP?

21. How can these barriers be mitigated?

22. What factors facilitate the implementation of VBP?

23. What enablers or drivers do you perceive in relation to VBP?

24. What lessons have you learned from your procurement projects?

25. If you could repeat these projects, what would you do differently? What aspects would you retain?
